# Hsa_circ_0060927 Is a Novel Tumor Biomarker by Sponging miR-195-5p in the Malignant Transformation of OLK to OSCC

**DOI:** 10.3389/fonc.2021.747086

**Published:** 2022-01-11

**Authors:** Siming Xu, Yuhan Song, Yanxiong Shao, Haiwen Zhou

**Affiliations:** ^1^ Department of Oral Mucosal Diseases, Shanghai Ninth People’s Hospital, College of Stomatology, Shanghai Jiao Tong University School of Medicine, Shanghai, China; ^2^ National Clinical Research Center for Oral Diseases, Shanghai, China; ^3^ Shanghai Key Laboratory of Stomatology & Shanghai Research Institute of Stomatology, Shanghai, China

**Keywords:** circRNA, high-throughput sequencing, OSCC, OLK, hsa_circ_0060927, ceRNA, miR-195-5p, TRIM14

## Abstract

**Objective:**

To investigate the clinical significance of differentially expressed circRNAs and candidate circRNAs in the transformation of oral leukoplakia (OLK) to oral squamous cell carcinoma (OSCC).

**Methods:**

We performed high-throughput circRNA sequencing in six cases of normal oral mucosal (NOM) tissues, six cases of OLK tissues, and six cases of OSCC tissues. Ten circRNAs with significant differential expression were verified by qRT-PCR. Enzyme tolerance assay and Sanger sequencing were performed on the screened target circRNA hsa_circ_0060927, and a qRT-PCR assay of hsa_circ_0060927 was performed in three tissues (24 cases in each group); this was followed by an ROC analysis. The ceRNA network was predicted using TargetScan and miRanda. MiR-195-5p and TRIM14 were selected as the downstream research objects of hsa_circ_0060927. The sponge mechanism of hsa_circ_0060927 was detected by AGO2 RIP. The interaction between hsa_circ_0060927 and miR-195-5p was verified by RNA pull-down assay and dual luciferase reporter gene assay. The expressions of hsa_circ_0060927, miR-195-5p, and TRIM14 were verified by normal oral epithelial primary cells and cell lines of LEUK1, SCC9, and SCC25. The hsa_circ_0060927 overexpressed plasmid and miR-195-5p mimics were constructed to transfection LEUK1 to detect the changes in cell proliferation, apoptosis, and migration.

**Results:**

The results of qRT-PCR validation were consistent with the sequencing results. Hsa_circ_0060927 is a true circRNA with trans-splicing sites. The expression of hsa_circ_0060927 increased in NOM, OLK, and OSCC. Overexpression of hsa_circ_0060927 enhanced the ability of cell proliferation and migration, and decreased cell apoptosis capacity. The prediction of ceRNA network suggested that hsa_circ_0060927 could regulate the target gene TRIM14 through sponging miR-195-5p. AGO2 RIP indicated that hsa_circ_0060927 had a sponge mechanism. RNA pull-down and dual luciferase reporter gene assay suggested that hsa_circ_0060927 interacted with miR-195-5p. Hsa_circ_0060927 was positively correlated with the expression of TRIM14, and could relieve the inhibition of miR-195-5p on TRIM14 to regulate cell proliferation, apoptosis, and migration of LEUK1 cells.

**Conclusion:**

Hsa_circ_0060927 acted as a potential key ceRNA to sponge downstream miR-195-5p and promote OLK carcinogenesis by upregulating TRIM14. Hsa_circ_0060927 was expected to be a molecular marker for the prevention and treatment of OLK carcinogenesis through the hsa_circ_0060927/miR-195-5p/TRIM14 axis.

## Introduction

Oral squamous cell carcinoma (OSCC) is the most common cancer in the oral maxillofacial region, and the incidence rate of OSCC has remained on an upward trend (1). Due to the high probability of regional lymph node metastasis and even distant metastasis, most OSCC patients are diagnosed as terminal cancer and have a poor prognosis (2, 3). Although molecular-targeted anti-tumor drugs have been widely applied clinically in recent years, the overall 5-year survival rate has reached up to 50%–60% (4). Therefore, it is essential to find out the reliable OSCC biomarkers for early diagnosis.

Oral leukoplakia (OLK) cannot be pathologically or clinically defined as any other diseases with white plaques or patches that cannot be rubbed off (5). It is well known that OLK is the most common oral potentially malignant disease clinically and may transform into OSCC (6). It has been reported that the rate of malignant transformation of OLK is between 0.13% and 34.0% (7, 8). Therefore, the focus of following research should be on the early detection of OSCC. However, it remains little known that the underlying molecular mechanism is responsible for the progression of OLK to OSCC.

Circular RNAs (circRNAs) are a large portion of endogenous non-coding RNAs with a covalently closed loop structure with no 5′ cap or 3′ polyadenylation tails (9, 10). Compared to linear RNAs, circRNAs are considered to be a new class of widespread, highly abundant, conserved, and stable noncoding RNAs (11). Recently, since circRNAs have been found to play a vital regulatory role in organisms, a number of circRNAs have attracted attention from a large group of researchers. It is demonstrated that many biological processes and molecular functions are affected by various circRNAs.

Despite the lack of protein coding potential, circRNAs could serve as a significant controlling gene in the physiopathologic process of OSCC in preliminary studies conducted by some researchers. For instance, Zheng et al. demonstrated that overexpression of circMDM2 acted as a poor prognostic factor and could accelerate the proliferation and glycolysis of OSCC through the circMDM2/miR-532-3p/HK2 axis *in vitro* and *in vivo* (12). The study of Yu et al. (13) indicated that the downregulation of circANTRL1 may play a role in radiosensitivity by sponging miR-23a-3p to promote PTEN expression. Liu et al. (14) also proved that circUHRF1 served as an oncogene in the progression of carcinogenesis and epithelial mesenchymal transformation of OSCC *via* the circUHRF1/miR-526b-5p/c-Myc/TGF-beta 1/ESRP1 pathway. These findings suggest that circRNA may play a role in oral cancer; nevertheless, the function and mechanism of circRNAs in oral premalignant lesion is still obscure. Our investigation provides new ideas to identify whether circRNAs participate in regulation in malignant transformation of OLK.

## Materials and Methods

### Tissue Specimens

OSCC, OLK, and normal oral mucosa (NOM) tissue specimens were used to perform high-throughput sequencing in a total of eighteen cases, with six samples in each group; all of those samples were selected from patients who underwent surgery and treatment in Shanghai Ninth People’s Hospital. The clinical manifestation of OLK and OLK canceration is shown in **Supplementary Figure S1**. The tissues used for qRT-PCR verification were obtained from 72 patients consisting of OSCC, OLK, and NOM groups. Each group has 24 patients. The clinical characteristics of the patients are shown in **Table 1**, including pathological information, OLK cell dysplasia, and OSCC staging. Normal gingival samples without inflammation and lesions in patients who underwent wisdom tooth extraction were obtained from oral and maxillofacial surgery and NOM tissues without inflammation, and lesions in patients who underwent plastic surgery were obtained from the Department of Plastic and Reconstructive Surgery. Diagnosis and classification of OSCC and OLK were confirmed by histopathological examination. Inclusion criteria were no immunotherapy (e.g., steroid therapy) and no induction differentiation therapy such as retinoic acid taken orally or used externally within 3 months. No patients had received previous radiotherapy or chemotherapy. Patients were older than 18 years old with no sex restriction in the selection criteria. The type of OLK patients is plaque, and all OSCC patients were diagnosed as primary cancer. In addition, there are two patients diagnosed with OSCC who had a previous history of OLK in the sequencing specimens (S3 and S6) and one in the validation specimens. All tissue specimens were frozen in liquid nitrogen immediately. All subjects provided written informed consent for experimental use of their tissues prior to surgery. Specimen information was recorded and data were encoded to protect patient privacy. All studies were authorized by the Ethics Committee of Shanghai Ninth People’s Hospital [No (2012). 21].

**Table 1:** Clinical characteristics of study subjects.

Variables	Samples used for sequencing (*n* = 18)			Samples used for validation (*n* = 72)		
	Normal (%)	OLK (%)	OSCC (%)	Normal (%)	OLK (%)	OSCC (%)
**Number**	6	6	6	24	24	24
**Age (mean ± SD)**	24.7 ± 4.8	53.5 ± 9.2	63.8 ± 15.6	32.5 ± 12.9	49.9 ± 11.8	59.5 ± 14.8
**Sex**						
** Male**	2 (33.3%)	2 (33.3%)	44 (66.7%)	10 (41.7%)	14 (58.3%)	13 (54.2%)
** Female**	4 (66.7%)	44 (66.7%)	2 (33.3%)	14 (58.3%)	10 (41.7%)	11 (45.8%)
**Sample site**						
** Tongue**	0	5 (83.3%)	5 (83.3%)	1 (4.2%)	15 (62.5%)	9 (37.5%)
** Buccal mucosa**	4 (66.6%)	1 (16.7%)	0	9 (37.5%)	6 (25%)	9 (37.5%)
** Gingiva**	1 (16.7%)	0	0	9 (37.5%)	2 (8.3%)	4 (16.7%)
** Others**	1 (16.7%)	0	1 (16.7%)	5 (20.8%)	1 (4.2%)	2 (8.3%)
**Dysplasia**						
** None**		0			2 (8.3%)	
** Mild**		3 (50%)			14 (58.3%)	
** Mild-moderate**		2 (33.3%)			2 (8.3%)	
**Moderate**		0			5 (20.8%)	
** Moderate-severe**		1 (16.7%)			0	
** severe**		0			1 (4.2%)	
**Stage**						
** Ⅰ**			0			2 (8.3%)
** Ⅰ–Ⅱ**			5 (83.3%)			9 (37.5%)
** Ⅱ**			1 (16.7%)			7 (29.1)
** Ⅱ–Ⅲ**			0			1 (4.2%)
** Ⅲ**			0			1 (4.2%)
** Others**			0			4 (16.7%)

### RNA Extraction and Quality Control

Total RNA was extracted from the experimental tissue samples using TRIzol (Life Technologies, Carlsbad, CA, USA). RNA concentrations and purity were tested by NanoDrop ND-1000 (Thermo Fisher Scientific, Waltham, MA, USA). Spectrophotometer OD260/OD280 values were used for RNA purity indexes. Quality control results indicated a range of OD260/OD280 between 1.8 and 2.1. Under the guidance of the manufacturer’s instructions, the rRNAs were eliminated by Ribo-Zero rRNA Removal Kits (Illumina, USA). RNA libraries were constructed by TruSeq Stranded Total RNA Library Prep Kit (Illumina, USA). The BioAnalyzer 2100 system (Agilent Technologies, USA) was applied for controlling the quality and quantified of libraries. CloudSeq Biotech (Shanghai, China) provided the high-throughput sequencing service. Libraries (10 pM) were denatured as single-stranded DNA molecules, captured on Illumina flow cells, amplified *in situ* as clusters and finally sequenced for 150 cycles on an Illumina HiSeq 4000 Sequencer (Illumina, San Diego, CA, USA).

### CircRNA Sequencing Data Analysis

To filter high-quality trimmed reads for analyzing circRNAs, Q30 was applied for quality controlling, and trim 3’ adaptors and low-quality reads were removed *via* cutadapt software (v1.9.3). The eligible trimmed reads were aligned to a reference genome/transcriptome with STAR software (v2.5.1b). The high-quality circRNAs were detected and identified by DCC software (v0.4.4). Identified circRNAs were annotated using circBase and circ2Traits databases. According to sequencing depth and degree of variation, we normalized the data and screened for altered circRNAs between OSCC and OLK using edgeR software (v3.16.5). The profile of differentially expressed circRNAs between OLK and OSCC was generated by Cluster and TreeView software. Based on the expression levels of all identified circRNAs in OLK and OSCC, the hierarchical clustering analysis proceeded and the significant differential circRNAs were selected. Unprocessed and analyzed sequencing data, after standardization, were uploaded to the National Center for Biotechnology Information Gene Expression Omnibus (GEO). The number of the dataset that was successfully uploaded to GEO is GSE131182 and GSE131568.

### Quantitative Real-Time RT-PCR

Reverse transcription was applied for total RNA by SuperScript III Reverse Transcriptase (Invitrogen, Carlsbad, USA). According to the manufacturer’s instructions, qRT-PCR reactions involved using qPCR SYBR Green master mix (Cloudseq, Shanghai, China) with QuantStudio 5 Real-Time PCR System (Thermo Fisher). Divergent primer pairs designed for target 10 circRNAs were selected and are summarized in **Table 2** using Primer 5.0. The reaction conditions were 95°C for 10 min and 40 cycles of 95°C for 10 s, 60°C for 15 s, and 72°C for 20 s, followed by 72°C for 5 min as elongation step. β-Actin, U6, and GAPDH were used as the normalization control for circRNA, miRNA, and mRNA, respectively.

**Table 2:** The primers used for quantitative real-time PCR (qRT-PCR) experiments.

Circular RNA	Primer type	Primer sequence (5’-3’)
chr14:81209419-81244390-	Forward	AGAAAGGCAGGAGCAGCTT
	Reverse	TCCAGCTGACCACGATGA
chr11:3752621-3774638-	Forward	CGGCCAAAGGCTTTACAA
	Reverse	CAAATCCTGCACCAAGCC
chr16:3900298-3901010-	Forward	GTGCTGGCTGAGACCCTAAC
	Reverse	AGCAGCATCTGGAACAAGGT
chr9:134381501-134381840+	Forward	GCTGGCCTTGGGAGGTTA
	Reverse	GGCCCACTGTCATCCAAG
chr19:34921481-34925873+	Forward	CCTGGCAGCTGATGTTCC
	Reverse	CTTGGCAACCTGTGCCTT
chr8:618598-624047-	Forward	AAAACGCTGCTGCTCCTG
	Reverse	TCTTTGGCTGGTCATGAGG
chr20:52773708-52788209-	Forward	GGCCACAGACAATGAGCC
	Reverse	AAATCGGCCAAGACCTCA
chr7:100410369-100410830-	Forward	CCAATATCATCCGCCTGG
	Reverse	CACCTCGCCAAACTCACC
chr17:29483001-29509683+	Forward	AGCAGCAGTTTGGCCACT
	Reverse	TGAGGCCGCTTATAACCAA
chr13:33306238-33320238+	Forward	ACTTGCTGCTGGGAGTGC
	Reverse	TGGCATATTTGGCTTGACG

### RNase R Assay and Sanger Sequencing

RNeasy MinElute Cleaning Kit (Qiagen) was used for RNase R assay, and total RNA was extracted from experimental tissues and then incubated with RNase R (5 U/mg; Epicentre Technologies). Next, the back-spliced junctions of hsa_circ_0060927 were verified by Sanger sequencing. Data analysis for relative expression of circRNAs was performed by 2^−∆∆Ct^ measurement.

### Identification and Validation of circRNAs

Step 1: A total of 389 circRNAs with a criterion of *p*-value < 0.05 and FC ≥ 2.0 were selected from the result of sequencing data. Step 2: According to the degree of the differentially expressed circRNAs, the characteristic of circRNAs and the correlation of disease annotation, ten circRNAs were elected from the 389 circRNAs selected in step 1. The ten circRNAs were validated by qRT-PCR. Step 3: Given that the aim was to detect whether there were molecules involved in the progress of OLK carcinogenesis, the comparison of differentially expressed circRNAs in normal versus OLK (N-K) and OLK versus OSCC (K-S) groups was intersected. Hsa_circ_0060927 was the intersection between the two groups and was also the most significant upregulated circRNA expression in K-S. Step 4: RNAase R and Sanger sequencing assay were used to validate the structure of hsa_circ_0060927. The candidate circRNA, hsa_circ_0060927, was further validated in an independent cohort that contained 24 OSCC, 24 OLK, and 24 healthy oral mucosa tissue samples, and ROC curve analysis was depicted after expanding the sample size for verification of hsa_circ_0060927. Cell localization and cell experiment were performed further to explore the expression and function of hsa_circ_0060927. Step 5: Mechanism analyses were performed to determine the importance of the hsa_circ_0060927/miR-195-5p/TRIM14 axis.

### Fluorescence *In Situ* Hybridization

The OLK tissue slices were used to perform fluorescence *in situ* hybridization (FISH) to locate hsa_circ_0060927 and miR-195-5p in OLK cells. In brief, FAM-labeled probes were specific to hsa_circ_0060927 and cy3-labeled probes were specific to miR-195-5p. Nuclei were stained by DAPI. All the procedures were conducted according to the manufacturer’s instructions (Asia-Vector Biotechnology, Shanghai, China). All images were acquired on an upright fluorescence microscope system (Nikon, Japan, Nikon DS-U3).

### Cell Line and Transfection

The Leuk1 cell line was derived from the University of Texas MD Anderson Cancer Center, USA (15–17), provided by Shanghai Key Laboratory of Stomatology affiliated to Shanghai Jiao Tong University School of Medicine. Leuk1 cells were cultured in a Defined keratinocyte-SFM (1×) medium. The medium was supplemented with 0.25% growth factor. SCC9 and SCC25 cell lines were purchased from the American Type Culture Collection (ATCC) and stored in Shanghai Key Laboratory of Stomatology. HEK293T cells were cultured in DMEM. The cells were identified as free from contamination by other cells and pathogens such as mycoplasma. Cells were incubated at 37°C in a cell incubator with a humidified atmosphere containing 5% CO_2_.

We constructed an hsa_circ_0060927-overexpressed vector. Briefly, the sequence with a full length of 1,106 bp was subcloned into a pCDH-CMV-MCS-EF1-copGFP-T2A-Puro vector (Asia-vector Biotechnology, Shanghai, China) to generate pLCDH-circ_0060927 constructs. The subcloned sequence containing a front circular frame (SA), a back circular frame (SD) of circRNA biogenesis, and a full length of hsa_circ_0060927 and 5’-ATTTAAATCGGATCCGGCCACACCCTCCCATCAAA-hsa_circ_0060927-ATCCTTCGCGGCCGCTCAGAACACAGCCTTTGTAGG -3’ was directly synthesized. The pLCDH-ciR empty vector was used as the control group. The procedure of transfection was conducted using Lipofectamine 2000 (Invitrogen) kits.

### Cell Viability

Cell proliferation was tested using Cell Counting Kit-8 (CCK-8) assay according to the manufacturer’s instruction. After 48 h of cell transfection, the cells were seeded in 96-well plates after cell counting, and the plates were cultured in the incubator. After cell attachment, 10 μl of CCK-8 solution (BBI Life Sciences, China) was added to each well on days 1–4. After incubation for 1 h, the absorbance was measured at 450 nm using a microplate reader (Bioteck, Epoch2). All results were expressed as the mean ± SD. Each experiment was proceeded at least three times independently.

### Cell Apoptosis Assay

Cell apoptosis assay was performed using an Annexin V–FITC/PI kit (Yeasen, Shanghai, China). After 48 h of cell transfection, cells were collected after trypsin digestion. Mixing resuspended cells using the Annexin V–FITC/PI kit was according to the manufacturer’s guidance. The cell concentration was 1 × 10^6^ cells/ml, and cell apoptosis assay was detected by flow cytometry (Beckman, FL, USA) after cell staining.

### Transwell Assay

After transfection for 48 h, LEUK1 cells were suspended in growth factor-free Defined keratinocyte-SFM. One hundred microliters of suspension cells was added to the Transwell chamber (Corning, NY, USA) after cell counting. Defined keratinocyte-SFM containing 0.5% growth factor was added to the lower chamber. After 24 s, nonmigratory cells were removed. The cells migrating through the membrane were counted under a microscope (Nikon, Tokyo, Japan) after fixing by formaldehyde and staining by 0.1% crystal violet.

### CircRNA–miRNA–Target Gene Network and Bioinformatic Analysis

CircRNA–miRNA-coding gene interactions were predicted by target prediction software, Target scan and miRanda, and the construction of the ceRNA network was used by the bioinformatics software Cytoscape (v2.8.0). In order to select the downstream target molecules in which hsa_circ_0060927 may regulate the process of OLK carcinogenesis, the miRNAs related to oral diseases that have been reported corresponded to the above predicted miRNAs. The combining capacity between circRNA and miRNA was predicted by bioinformation software.

The functional mechanism analysis of the differentially expressed circRNAs between OLK and OSCC was predicted by Gene Ontology (GO) and Kyoto Encyclopedia of Genes and Genomes (KEGG) analysis. GO terms were screened according to the source gene with significantly different circRNAs and GO annotation list. The *p*-value indicates the statistical significance of GO terms enrichment in the host genes using Fisher’s exact test (*p*-value < 0.05 is recommended). KEGG analysis was used to annotate host genes of differentially expressed circRNAs using the KEGG database. The Fisher *p*-value denotes the significance of each pathway involved, and *p* < 0.05 was deemed to indicate statistically significant differences.

### AGO2 RIP

RIP assays were performed in OLK tissues. Resuspended cells were collected from tissue homogenate. RNA lysis buffer was used to lyse the cells. Then, the cells were incubated in RIP immunoprecipitation buffer containing magnetic beads conjugated with human anti-Argonaute2 (AGO2) antibody (Millipore, USA) or negative control Rabbit IgG (Millipore, USA). Proteinase K was added to the RIP sample and incubated at 55°C for 30 min. Then, immunoprecipitated RNA was isolated and analyzed by qRT-PCR to quantify the enrichment of hsa_circ_0060927.

### RNA Pull Down

Biotin-labeled hsa_circ_0060927 probe (positive probe) and oligo probe (GenePharma, China) (negative probe) were synthesized. OLK tissues were lysed with lysis buffer and incubated with specific hsa_circ_0060927 probes. Then, OLK tissues were lysed with lysis buffer and incubated with probe-coated beads at 4°C overnight. The beads were washed, the RNA complexes were extracted with TRIzol (Life Technologies, Carlsbad, CA), and qRT-PCR was performed to detect miR-195-5p.

### Dual-Luciferase Reporter Assay

The wild-type hsa_circ_0060927 (hsa_circ_0060927-WT) vector, which contained miR-195-5p-specific binding sites, and the mutant fragment (hsa_circ_0060927-Mut), which contained the mutant miR-195-5p binding sites, were constructed by annealing double-stranded DNA and inserting them into the pSI-Check2 vector (Cloudseq Biotech, Inc.) (18). Lipofectamine 3000 (Invitrogen) reagent was utilized for co-transfection of miR-195-5p mimic/NC and the above-mentioned plasmids to the 293T cells. After 48 h of transfection, luciferase activity assay was conducted according to the description of the manufacturer.

### Statistical Analysis

Statistical analysis proceeded using SPSS 19.0 (SPSS, Chicago, IL, USA) and GraphPad Prism version 8.0 software. *t*-test was performed according to actual conditions. Statistical significance of difference between groups was determined by unpaired *t*-test and one-way ANOVA (*p < 0.05, **p < 0.01, ***p < 0.001, ****p < 0.0001). Correlations were analyzed using Pearson’s linear correlation analysis. *p* < 0.05 was considered statistically significant. Student’s receiver operating characteristic (ROC) was performed to assess diagnostic and prognostic values of hsa_circ_0060927. Quantitative data were shown as mean ± standard error of the mean (SEM), and all the data were obtained through three times repetition at least.

## Results

### Expression Profile and Characteristic of Differentially Expressed circRNAs

**Figure 1:**
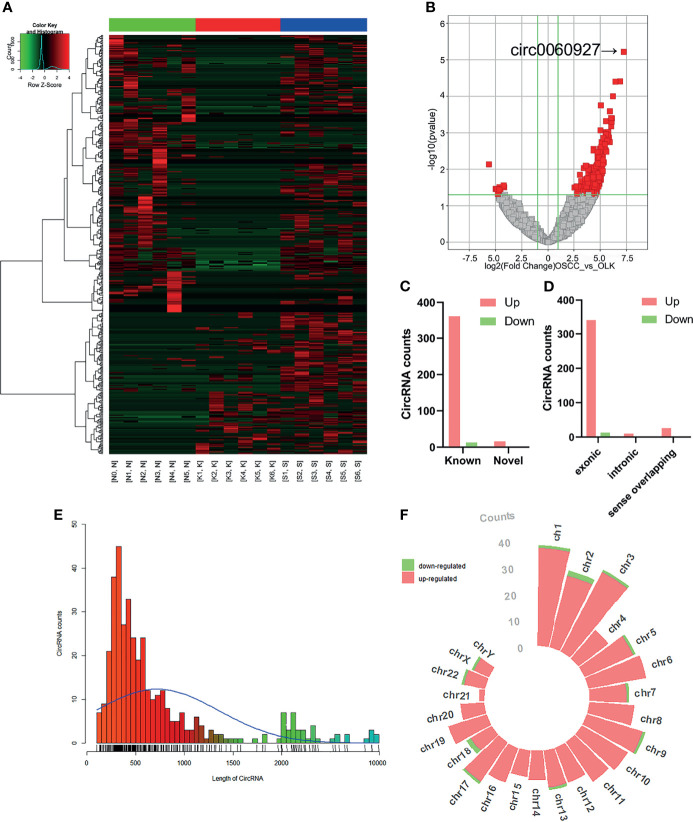
Expression profiles and distribution characteristics of circRNAs in oral squamous carcinoma cell (OSCC) versus oral leukoplakia (OLK) tissues. **(A)** Hierarchical clustering analysis showed circRNA expression profiles that were different between OSCC, OLK, and NOM tissues. Hierarchical clustering analysis for 18 samples in a disease group (S1, S2, S3, S4, S5, and S6) (K1, K2, K3, K4, K5, and K6) and a control group (N0, N1, N2, N3, N4, and N5). **(B)** Volcano plot of significantly dysregulated circRNAs in OLK tissues. Red, eligible circRNAs (logFC ≥ 2.0, *p*-value < 0.05). **(C)** Filtered circRNAs were classified by whether they were newly discovered. **(D)** Significantly differentially expressed circRNAs were divided into three types according to host gene structure: exonic, intronic, and sense overlapping circRNA. Red, upregulated; green, downregulated circRNA. **(E)** Classification according to circRNA length. **(F)** Chromosome distribution of upregulated and downregulated significantly differentially expressed circRNAs.

The differential circRNAs among OSCC, OLK, and normal groups were compared. Hierarchical clustering diagrams showed relationship among the samples (**Figure 1A**). Since the carcinogenesis of OLK is mainly discussed in this manuscript, we compared the differentially expressed circRNAs between OLK and OSCC, and a volcanic plot was painted with statistical criteria determined by fold change (FC) and *p*-value (**Figure 1B**). Finally, 389 significant dysregulated circRNAs were identified, with 376 markedly upregulated and 13 downregulated circRNA s in OSCC compared with OLK.

Among the 389 dysregulated circRNAs, 16 (4.1%) new circRNAs were identified with the rest found (95.9%) in the circRNA database (19). Most were known and exonic circRNAs (**Figures 1C, D**). The lengths of these circRNAs were concentrated in approximately 500 bp (**Figure 1E**). The circRNAs were located on nearly all human chromosomes: the 22 autosomes and X chromosome (**Figure 1F**).

### Screening and Validation of circRNAs

**Figure 2:**
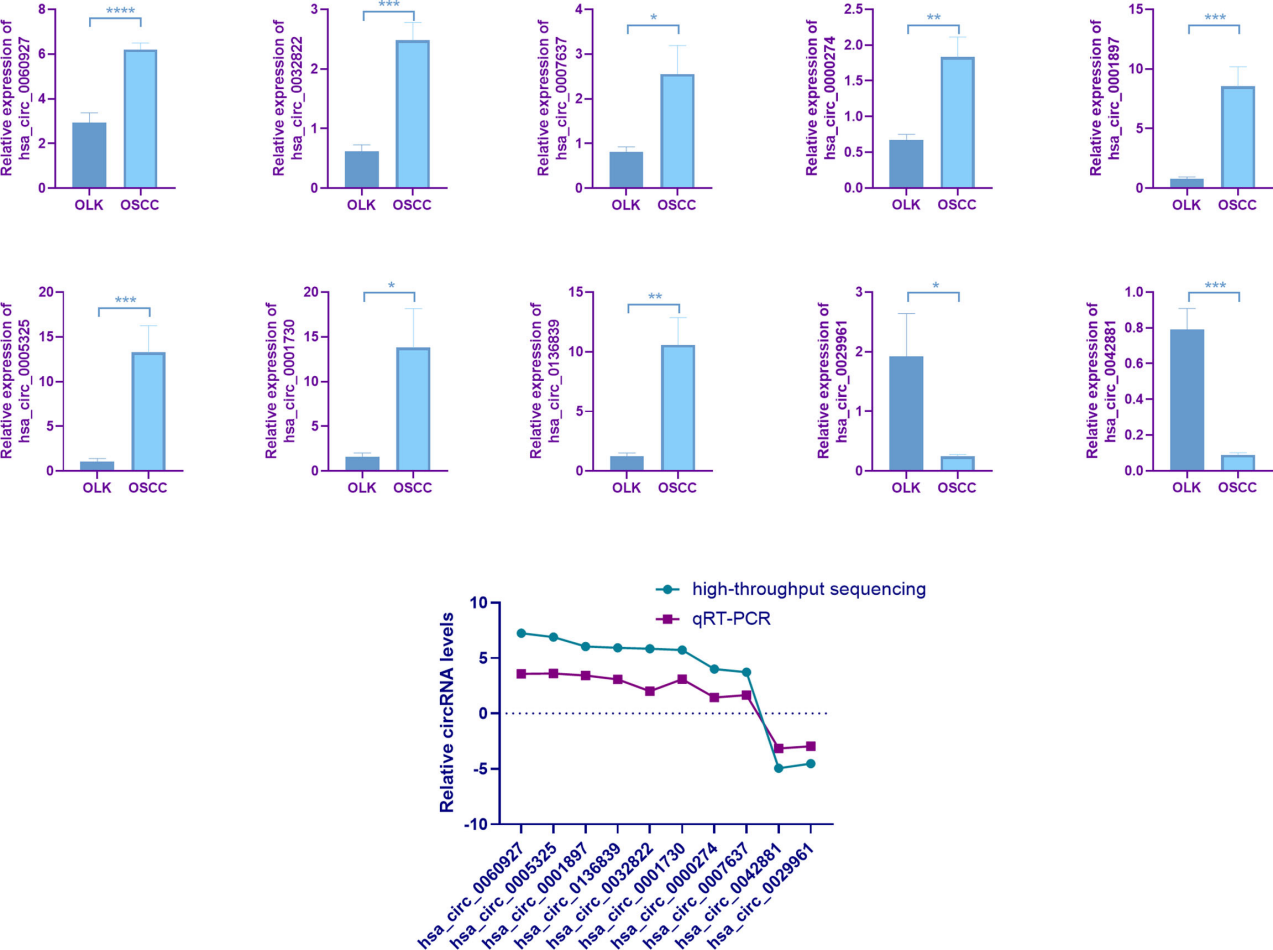
Quantitative real-time PCR (qRT-PCR) verification. Six pairs of tissues from patients with oral leukoplakia (OLK) and oral squamous carcinoma cell (OSCC) were used for validating by qRT-PCR. Ten selected circRNAs showed relative expression in OLK compared with OSCC. All the ten circRNAs, has_circ_0060927 (*p* < 0.0001), has_circ_0032822 (*p* < 0.001), has_circ_0007637 (*p* < 0.05), has_circ0000274 (*p* < 0.01), has_circ_0001897 (*p* < 0.001), has_circ_0005325 (*p* < 0.001), has_circ_0001730 (*p* < 0.05), has_circ_0136839 (*p* < 0.01), has_circ_0029961 (*p* < 0.05), and has_circ_0042881 (*p* < 0.001), exhibited the same expression trend toward the sequencing results. The results of ten verified circRNAs of high-throughput sequencing and qRT-PCR were exhibited together as logFC (fold change) in the OLK group. (*p < 0.05, **p < 0.01, ***p < 0.001, ****p < 0.0001).

Top eight upregulated and two downregulated circRNAs were selected for verification by qRT-PCR. Validation was first performed on the six pairs of OSCC and OLK tissue (**Supplementary Table S1**). Of the ten verified circRNAs, the results were consistent with the circRNA sequencing (**Figure 2**).

**Figure 3:**
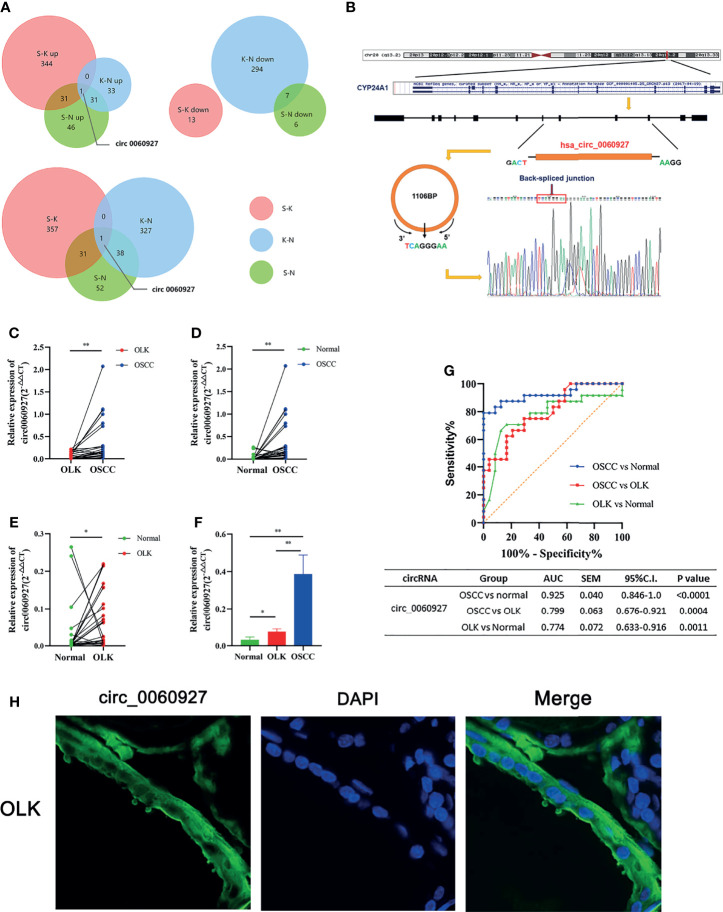
Further validation and visualization analysis of has_circ_0060927. **(A)** Wynn diagram of differentially expressed circRNAs in OSCC-OLK (S-K), OLK-Normal (K-N), and OSCC-Normal (S-K). Red, S-K; blue, K-N; Green, S-N. **(B)** The ring formation mechanism and back spliced site of has_circ_0060927. The Sanger sequencing results of circ_0060927 demonstrate that it was a real circRNA with the specific back splice sites (TCAGGGAA). **(C)** The relative expression in OLK vs. OSCC, **(D)** normal vs. OSCC, **(E)** normal vs. OLK, and **(F)** normal vs. OLK vs. OSCC in the independent cohort with 24 tissues in each group. (*p < 0.05, **p < 0.01). **(G)** ROC analysis of circ_0060927 in OSCC vs. normal, OSCC vs. OLK, and OLK vs. normal group. The area under the curve (AUC) showed that circ_0060927 could be a potential diagnosis and prediction biomarker in the OSCC vs. OLK group followed by the OSCC vs. normal group. **(H)** FISH analysis of has_circ_0060927.

In order to better compare the differential circRNAs in OSCC, OLK,and NOM groups, we performed an intersection analysis on the upregulated, downregulated, and total differentially expressed gene of the three groups respectively; Venn diagrams were drawn (**Figure 3A**). The results showed that there were more intersections between the OSCC-OLK group and OSCC-Normal group, while the OLK-Normal group had fewer intersections with the other two groups. Interestingly, hsa_circ_0060927 stood out from the intersection as a continuous differential variable in the progress of OLK carcinogenesis. Since circRNA is not sensitive to nuclease, enzyme-resistant test and Sanger sequencing were performed to verify hsa_circ_0060927. The results showed that hsa_circ_0060927 was a real circRNA with a specific back-splice junction site (TCAGGGAA). We further performed a visualization analysis of hsa_circ_0060927, which was derived from CYP24A1 and consisted of 12 exons and 11 introns (**Figure 3B**).

### Further Validation and Cell Localization of hsa_circ_0060927

To further verify the expression of hsa_circ_0060927 and its credibility as an early biomarker of oral cancer, three groups of independent samples consisting of 24 OSCC, 24 OLK, and 24 healthy tissues were subjected to qRT-PCR, with the results the same as before (**Figures 3C**–**F**). The expression of hsa_circ_0060927 was increasing in NOM, OLK, and OSCC, and its expression was significantly upregulated in the canceration of normal mucosa and OLK, suggesting that hsa_circ_0060927 may be a potential biomarker for promoting oral cancer progression. Based on the expression of hsa_circ_0060927 to draw the ROC curve, it can be seen that the area under curve (AUC) was large, which means high diagnostic sensitivity and specificity (**Figure 3G**). In these three comparisons, hsa_circ_0060927 was eligible to be used as a biomarker for diagnosis and prediction in the canceration progression of normal mucosa and OLK with certain credibility. Fluorescence *in situ* hybridization (FISH) revealed that hsa_circ_0060927 localized in cytoplasm of OLK (**Figure 3H**).

### Functional Enrichment Analysis of hsa_circ_0060927

One of the functions of circRNAs is that circRNAs could promote the expression of host gene (20–22). GO covers three domains: biological processes (BP), cell components (CC), and molecular function (MF). Among 389 differentially expressed genes in OSCC versus OLK tissues, 376 were upregulated altered circRNAs. Since upregulated circRNAs accounted for the majority of differentially expressed circRNAs, we mainly classified and counted the GO items including the target genes of the 376 upregulated circRNAs. KEGG analysis identified functional pathways through mapping relevant circRNAs to the KEGG pathway database. We selected top 10 terms for each category based on the fold enrichment count (**Supplementary Figure S2**).

**Figure 4:**
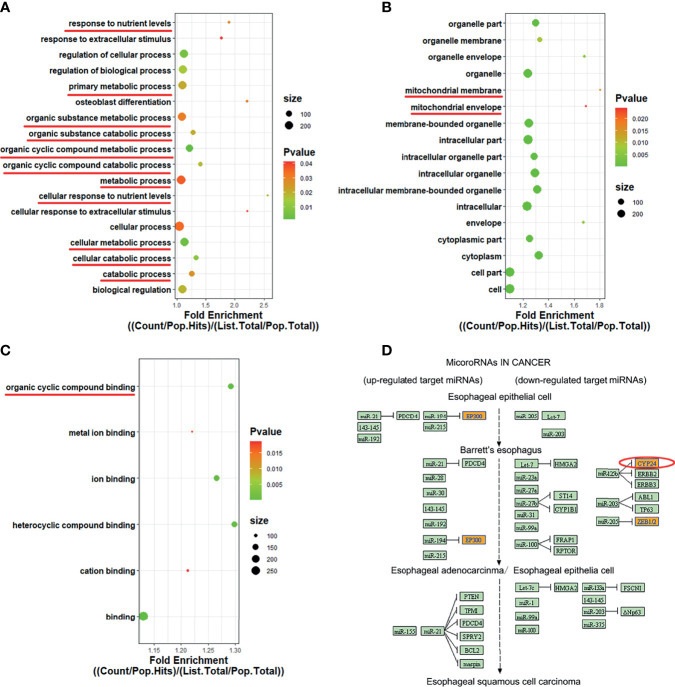
GO enrichment and KEGG pathway analysis of has_circ_0060927. **(A–C)** GO terms were exhibited in BP, CC, and MF bubble diagram, respectively. **(D)** KEGG pathway involved in has_circ_0060927.

The GO and KEGG pathways involved in hsa_circ_0060927 are summarized in **Figure 4**. Cytochrome P450 family 24 subfamily A member 1 (CYP24A1), the source gene of hsa_circ_0060927, is located on chromosome 20 (23). GO analysis showed that, in terms of BP (**Figure 4A**), CYP24A1 was involved in “cellular regulation of organic matter metabolism and decomposition” (GO: 0044237, GO: 1901575), “trophic level cellular response” (GO: 0031669), and “response to extracellular stimuli” (GO: 0009991). As regards CC (**Figure 4B**), “endometrium organelles” (GO: 0043231), “cytoplasmic part” (GO: 0044444), and “mitochondrial membrane” (GO: 0031966) were the most common enrichment categories. As for MF (**Figure 4C**), “organic ring compound binding” (GO: 0097159) and “ion binding” (GO: 0043167) were mainly enriched. KEGG analysis showed that the target gene CYP24A1 was involved in the pathway “miRNA in tumors” (Hsa05206), as shown in **Figure 4D**, which provided a basis for us to further predict the interaction between downstream miRNA and hsa_circ_0060927.

### Overexpression of hsa_circ_0060927 Promotes LEUK1 Cell Proliferation and Migration and Inhibits Cell Apoptosis

**Figure 5:**
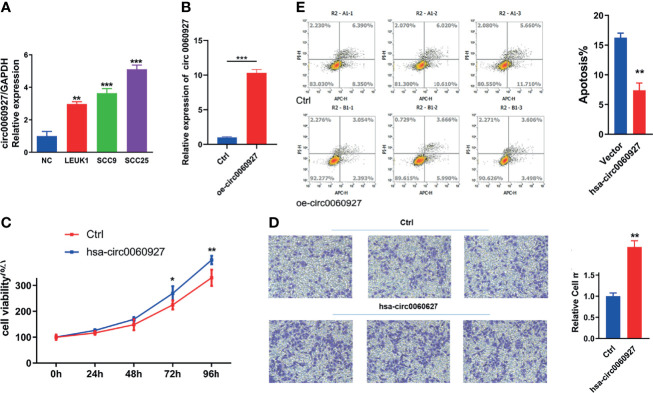
Function analysis of has_circ_0060927. **(A)** The expression of has_circ_0060927 in NOM, LEUK1, SCC9, and SCC25 cells. **(B)** The expression change of has_circ_0060927 after transfection withhas_circ_0060927 overexpression plasmid. **(C)** CCK-8 assay. **(D)** Apoptosis assay. **(E)** Transwell assay. (**p < 0.01, ***p < 0.001).

The expression of hsa_circ_0060927 showed an increasing trend in normal oral mucosal keratinocytes, LEUK1, SCC9, and SCC25 cells (**Figure 5A**), which was the same as that in normal oral mucosal epithelium, oral leukoplakia, and oral squamous cell carcinoma tissues. Leuk1 cells were transfected with empty plasmid and hsa_circ_0060927 plasmid, respectively. The qRT-PCR results showed that the expression level of hsa_circ_0060927 in the transfected hsa_circ_0060927 plasmid group was significantly increased, indicating that the hsa_circ_0060927 plasmid was successfully transfected (**Figure 5B**). After transfection of LEUK1 with hsa_circ_0060927, the cell proliferation ability of LEUK1 cells at 24 h,72 h, and 96 h was detected. The CCK-8 assay demonstrated that overexpression of hsa_circ_0060927 significantly promoted the proliferation viability of LEUK1 cells (**Figure 5C**). We further explored whether hsa_circ_0060927 has an effect on the migration of LEUK1 cells by transwell assay. As a result, the cell migration abilities of LEUK1 cells were significantly enhanced after overexpression of hsa_circ_0060927 (**Figure 5D**). Next, we investigated the apoptosis abilities of LEUK1 cells by flow cytometry. Double staining with Annexin V and PI indicated that hsa_circ_0060927 overexpression significantly inhibited cell apoptosis at 48 h post transfection with hsa_circ_0060927 plasmid (**Figure 5E**). These results showed that hsa_circ_0060927 contributed to LEUK1 cell proliferation, apoptosis, and motility *in vitro*.

### Hsa_circ_0060927 Functions as a Sponge for miR-195-5p

**Figure 6:**
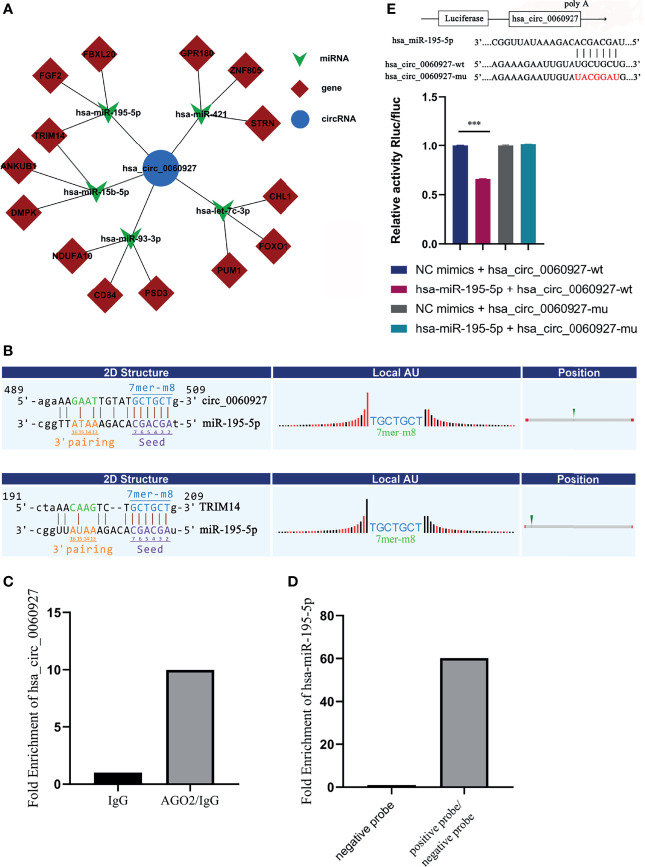
Hsa_circ_0060927 functioned as a sponge for miR-195-5p. **(A)** ceRNA network predicted downstream miRNA and mRNA of hsa_circ_0060927. **(B)** The online software revealed the potential binding site between hsa_circ_0060927/miR-195-5p and TRIM14/miR-195-5p. **(C)** RIP assay. **(D)** RNA pull-down assay. **(E)** Dual luciferase reporter assay showed luciferase activities of hsa_circ_0060927 reporter gene after co-transfection with miR-195-5p or NC mimic; *n* = 3. (***p < 0.001).

Given that circRNAs may act as a molecular sponge to relieve the inhibition of miRNA on mRNA, mRNA expression (24) was affected, and the top 5 miRNAs and top 3 mRNAs that could match hsa_circ_0060927 were predicted by bioinformation software (**Figure 6A**). Among the 5 miRNAs, miR-195-5p and miR-15b-5p, which are known to be involved in regulating multiple oncogene expressions, have been shown to play a significant role in OSCC (25, 26). The result of prediction indicated that the form of binding sites between miR-195-5p, miR-15b-5p, and hsa_circ_0060927 was both 7mer-m8, and the binding sites of miR-195-5p/hsa_circ_0060927 and miR-15b-5p/hsa_circ_0060927 were identical, both of which were GCTGCT (**Figure 6B**). Downstream target genes of miR-195-5p and miR-15b-5p were predicted. It has been reported that TRIM14 is the target gene of miR-195-5p and miR-15b-5p, and could also act as a vital biomarker in the diagnosis of OSCC (25–27). According to the prediction of the binding score between circRNA and miRNA, the binding ability of miR-195-5p was the strongest. Studies showed that TRIM14 could act as an oncogene under the regulation of miR-195-5p (28). Therefore, the miR-195-5p/TRIM14 axis was selected as the subject of the subsequent study of hsa_circ_0060927.

RNA immunoprecipitation (RIP) assay was then performed in OLK tissues to determine the association between hsa_circ_0060927 and AGO2. AGO2 protein was a key molecule for circRNA to sponge miRNA.

The quantitative real-time PCR results showed that the expression of hsa_circ_0060927 pulled down with anti-AGO2 antibodies was significantly higher compared to the anti-IgG group (**Figure 6C**), suggesting that hsa_circ_0060927 could directly target AGO2. This means that hsa_circ_0060927 has the function of ceRNA. On the other side, RNA pull-down assay showed that the relative abundance of miR-195-5p treated with biotinylated hsa_circ_0060927-positive probe was significantly upregulated, implying that miR-195-5p could combine to hsa_circ_0060927 (**Figure 6D**). In order to further study the interaction between hsa_circ_0060927 and miR-195-5p, the dual luciferase reporter gene system was used to preliminarily confirm the interaction between the two. The dual luciferase reporter gene system refers to the experiment of verifying target genes by detecting the luciferase activity. The results demonstrated that overexpression of miR-195-5p could remarkably reduce the luciferase activity of the vector containing the wild-type hsa_circ_0060927 sequence but not the luciferase activity of the vector with mutant binding sites (**Figure 6E**). These results suggested that hsa_circ_0060927 could bind to AGO2 and act as a miR-195-5p sponge.

### Hsa_circ_0060927 Plays an Oncogenic Role in LEUK1 Cells *via* the miR-195-5p/TRIM14 Axis

**Figure 7:**
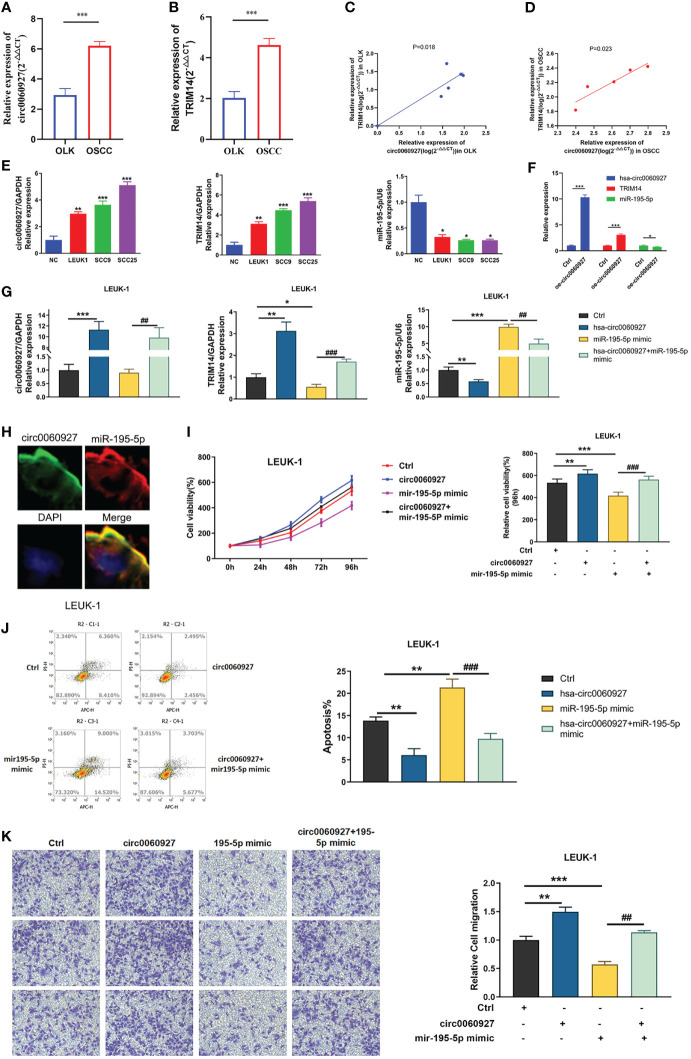
hsa_circ_0060927 played an oncogenic role in LEUK1 cells *via* the miR-195-5p/TRIM14 axis. Bioinformatics prediction and Luciferase reporter assay analysis. **(A)** The expression of miR-195-5p and **(B)** TRIM14 in OLK (*n* = 6) and OSCC (*n* = 5). **(C)** Positive correlation between hsa_circ_0060927 and TRIM14 in OLK and **(D)** OSCC. **(E)** The expression of hsa_circ_0060927, TRIM14, and miR-195-5p in NOM, LEUK1, SCC9, and SCC25 cells. **(F)** The expression alteration of hsa_circ_0060927, TRIM14, and miR-195-5p after transfection of overexpressed- hsa_circ_0060927 (oe- circ_0060927) in LEUK1 cells. **(G)** The expression level of hsa_circ_0060927, miR-195-5p, and TRIM14 after overexpression of the hsa_circ_0060927 plasmid and miR-195-5p mimics. **(H)** The co-localization of hsa_circ_0060927 and miR-195-5p in OLK. **(I)** CCK-8 assay. **(J)** Apoptosis assay. **(K)** Transwell assay.

Additionally, qRT-PCR analysis was performed to figure out the correlation between TRIM14 and hsa_circ_0060927. Results showed that the expression trend of TRIM14 was identical with hsa_circ_0060927 in OLK and OSCC (**Figures 7A, B**), and we observed a positive correlation between hsa_circ_0060927 and TRIM14 expression in OLK and OSCC tumor tissues (**Figures 7C, D**). In addition, the expression levels of hsa_circ_0060927/TRIM14 in cells were consistent with the expression in tissues. The expression trends of TRIM14 and hsa_circ_0060927 were consistent and rose in normal, LEUK1, SCC9, and SCC25 cells gradually. We also verified the expression level of miR-195-5p, and it was shown that in LEUK1, SCC9, and SCC25 cells, the expression trend of miR-195-5p was opposite to that of TRIM14 and hsa_circ_0060927, showing a decreasing expression (**Figure 7E**).

After overexpression of hsa_circ_0060927 in LEUK1 cell lines, the expression of hsa_circ_0060927/miR-195-5p/TRIM14 was detected, and the results showed that the expression of TRIM14 was significantly increased, while the expression of miR-195-5p was significantly decreased (**Figure 7F**). Additionally, FISH assay revealed that hsa_circ_0060927 and miR-195-5p were co-localized in the cytoplasm (**Figure 7H**). Based on the results showing a tumor-promoting effect of hsa_circ_0060927 on LEUK1 cells, the study next evaluated whether the effects of hsa_circ_0060927 were mediated by the miR-195-5p/TRIM14 axis. Therefore, the cells were co-transfected with oe-hsa_circ_0060927 and miR-195-5p mimics (**Figure 7G**). After increasing the expression of miR-195-5p, the proliferative (**Figure 7I**), apoptosis (**Figure 7J**), and invasive (**Figure 7K**) capabilities of LEUK1 cells caused by the overexpression of hsa_circ_0060927 were restored. These results showed that hsa_circ_0060927 contributed to LEUK1 cell proliferation, apoptosis, and motility through the miR-195-5p/TRIM14 axis.

## Discussion

Oral cancerization is a multistep process impressed by genetic modifications leading the cells to proliferate in an uncontrollable way (7, 29). Up to now, there is a limited understanding of the underlying molecular mechanisms accounting for the progression of oral pre-malignant lesion to malignant tumor.

At present, most of the topic of circRNA in the area of stomatology is normal tissue development to cancer. In this study, we performed high-throughput circRNA sequencing in OLK tissues and OSCC tissues and obtained the expression profile of 21605 circRNAs in OLK. Then, significantly changed circRNAs between OLK and OSCC were screened. These circRNAs may become potential biomarkers and therapeutic targets for the diagnosis of OLK carcinogenesis. Among the circRNAs of OLK versus OSCC, hsa_circ_0060927 was found to be the most significantly differentially expressed circRNA in OLK tissues and cell lines, and the expression of hsa_circ_0060927 was gradually increasing in the progress of NOM-OLK-OSCC tissues and cell lines. In addition, hsa_circ_0060927 was confirmed to be a real circRNA with special back-spliced junction by RNase R and Sanger sequence assay. ROC analysis showed that hsa_circ_0060927 had high sensitivity and specificity in the diagnosis of oral mucosa carcinogenesis. It suggested that hsa_circ_0060927 may have diagnostic value for oral mucosa carcinogenesis.

Currently, many studies have shown that the exon circRNA and annotated trans-spliced circRNAs are mainly located in the cytoplasm (30–32). In addition, most stable circRNAs can competitively bind with mRNA to regulate gene expression, which was all carried out in the cytoplasm (30, 33). Our FISH results showed that hsa_circ_0060927 was located in the cytoplasm of LEUK1 cells. The first reported circRNA sponge mechanism model, CIRS-7 (Circular RNA sponge for miR-7), was localized in the cytoplasm and highly expressed in human and mouse brain cells, and its RNA sequence contained more than 60 miR-7 targeting binding sites (9). Circular RNA_LARP4, which could be used as a sponge to adsorb miR-424-5p and regulate LATS1 expression to inhibit the proliferation and invasion of gastric cancer cells, was also localized in the cytoplasm (34). It was reported that hsa_circ_0060927 may serve as a potential diagnostic biomarker for human colorectal and uterine leiomyomas (35, 36). Based on the characteristics of circRNAs in the cytoplasm in literatures, hsa_circ_0060927 may have rich biological functions. The host gene of hsa_circ_0060927, CYP24A1, was a mitochondrial enzyme responsible for neutralizing active vitamin D (37). The enriched functional pathways suggested that CYP24A1 was involved in nutrient metabolism, mitochondrial membrane, and the binding of some cyclic compounds. Studies showed that CYP24A1 could act as an oncogene and was upregulated in a variety of tumors, such as human breast, lung, colon, and ovarian tumors (38). Zeljic et al. (39) suggested that gene polymorphism of CYP24A1 might affect oral cancer susceptibility. It was reported that circRNA could play a biological role by promoting the expression of source genes, but the specific mechanism of hsa_circ_0060927 inducing oral cancer remains to be further explored. Next, a series of functional experiments demonstrated that overexpression of hsa_circ_0060927 significantly promoted the proliferation and migration of LEUK1 cells and inhibited the apoptosis of LEUK1 cells, revealing its function as an oncogene. It can be speculated that hsa_circ_0060927 plays a key role in the malignant progression of OLK.

The study of Kjems et al. (24) found that circRNAs can act as an “miRNA sponge” in the manner of adsorbing miRNA by combining AGO2 protein. The RIP assay was used to confirm that hsa_circ_0060927 has a sponge adsorbing mechanism. On the basis of hsa_circ_0060927, we first predicted circRNA–miRNA–mRNA interactions through bioinformation software and constructed ceRNA networks. We further confirmed that hsa_circ_0060927 could directly interact with miR-195-5p, one of the predicted targets, by using RNA pull-down and dual luciferase reporter assays, which was consistent with prediction results. miR-195-5p was the miRNA with a higher binding score with hsa_circ_0060927 in the predicted results. Meanwhile, miR-195-5p had an inhibitory effect on squamous cell lung cancer, colorectal cancer, breast cancer, etc. (40–42). After we determined that miR-195-5p could be adsorbed by hsa_circ_0060927, the next focus was to search for its effector target genes. In a similar way, we predicted several of the most likely potential downstream genes from the target gene prediction software. Expression level detection analysis demonstrated that TRIM14 was positively correlated with the expression of hsa_circ_0060927 in both tissues and cells. However, the expression of miR-195-5p was opposite to the level of hsa_circ_0060927. It suggested that hsa_circ_0060927 may promote the expression of TRIM14 by relieving the inhibition of miR-195-5p. In addition, overexpression of hsa_circ_0060927 relieved partial miR-195-5P inhibitory effect on TRIM14, thus promoting the carcinogenic effect of TRIM14. The results of *in vitro* experiments showed that the effect of increased LEUK1 cell proliferation and migration caused by hsa_circ_0060927 overexpression was offset by the addition of miR-195-5p; similarly, the effect of LEUK1 cell apoptosis was also offset by the increase of miR-195- 5p. Our results indicated that hsa_circ_0060927 serves as an oncogene by sponging miR-195-5p in OLK.

It was reported that TRIM14 was a direct target of miR-195-5p, and miR-195-5p could specifically bind to the 3 ‘-UTR of TRIM14 to inhibit its expression. The targeted inhibition of miR-195-5p on TRIM14 was reported in OSCC and gastric cancer (26, 27). According to the reports, TRIM14 was a mitochondrial adapter that promoted innate immune signal transduction and played an important role in antiviral defense (43). In previous studies, TRIM14 was considered as an oncogene in a variety of cancers, and its high expression was associated with cancer cell proliferation, invasion, and poor prognosis of patients (44). Overexpression of TRIM14 promoted tongue squamous cell carcinoma by activating the NF-kb signaling pathway (43). However, the function of miR-195-5p and TRIM14 was still obscured in the process of oral pre-cancer transformation. Our study indicated that hsa_circ_0060927 exerted a carcinogenic effect through the hsa_circ_0060927/miR-195-5p/TRIM14 axis. The pathway in which the hsa_circ_0060927/miR-195-5p/TRIM14 axis plays a role in OLK canceration will be further discussed in future experiments.

In conclusion, our study profiled and validated altered circRNAs between OSCC, OLK, and normal tissue. Our results revealed that hsa_circ_0060927 might serve as a great diagnosis biomarker in OLK and OSCC, and might play critical roles in the development of OLK to OSCC through the hsa_circ_0060927/miR-195-5p/TRIM14 axis. Looking for valuable malignant transformation of OLK biomarkers not only might help diagnose OSCC at the very early stage, but also might contribute to stratify the OLK patients with low risk and high risk for progression into OSCC, which will provide important guidance for personalized and precision therapy.

## Data Availability Statement

The datasets presented in this study can be found in online repositories. The names of the repository/repositories and accession number(s) can be found at: NCBI, GSE131182, and GSE131568.

## Ethics Statement

The studies involving human participants were reviewed and approved by the Ethics Committee of Shanghai Ninth People’s Hospital. The patients/participants provided their written informed consent to participate in this study.

## Author Contributions

HZ conceptualized the study. SX, YHS, and YXS developed the methodology. SX was in charge of the software. SX, YHS, and YXS validated the study. SX, YHS, and YXS conducted the formal analysis and the investigation. HZ provided the resources. SX, YHS, and YXS conducted the data curation. SX wrote and prepared the original draft. SX, YHS, YXS, and HZ wrote, reviewed, and edited the manuscript. SX and HZ conducted the visualization. HZ supervised the study. All authors contributed to the article and approved the submitted version.

## Funding

This work was supported by the National Key R&D Program of China (2017YFC0840100 and 2017YFC0840110) and Biobank Program of Shanghai Ninth People’s Hospital (YBKA201912).

## Conflict of Interest

The authors declare that the research was conducted in the absence of any commercial or financial relationships that could be construed as a potential conflict of interest.

## Publisher’s Note

All claims expressed in this article are solely those of the authors and do not necessarily represent those of their affiliated organizations, or those of the publisher, the editors and the reviewers. Any product that may be evaluated in this article, or claim that may be made by its manufacturer, is not guaranteed or endorsed by the publisher.

## Supplementary Material

Supplementary Figure S1 The clinical lesion manifestation of OLK and malignant transformation of OLK. **(A)** The clinical feature of OLK, OLK can occurs in any part of the oral mucosa, including tongue, buccal, palate and gingiva etc. **(B)** The clinical manifestation of malignant transformation of OLK.

Supplementary Figure S2 GO enrichment and KEGG pathway analysis. **(A–C)** Top 10 GO terms were exhibited in BP, CC and MF bubble diagram respectively. **(D)** Top ten KEGG pathway involved in differentially expressed circRNAs.
