# Supplementary figures and images for: Hsa_circ_0060927 Is a Novel Tumor Biomarker by Sponging miR-195-5p in the Malignant Transformation of OLK to OSCC
Source: Front Oncol. 2022 Jan 11;11:747086. doi: 10.3389/fonc.2021.747086 (PMC8786726; doi:10.3389/fonc.2021.747086)

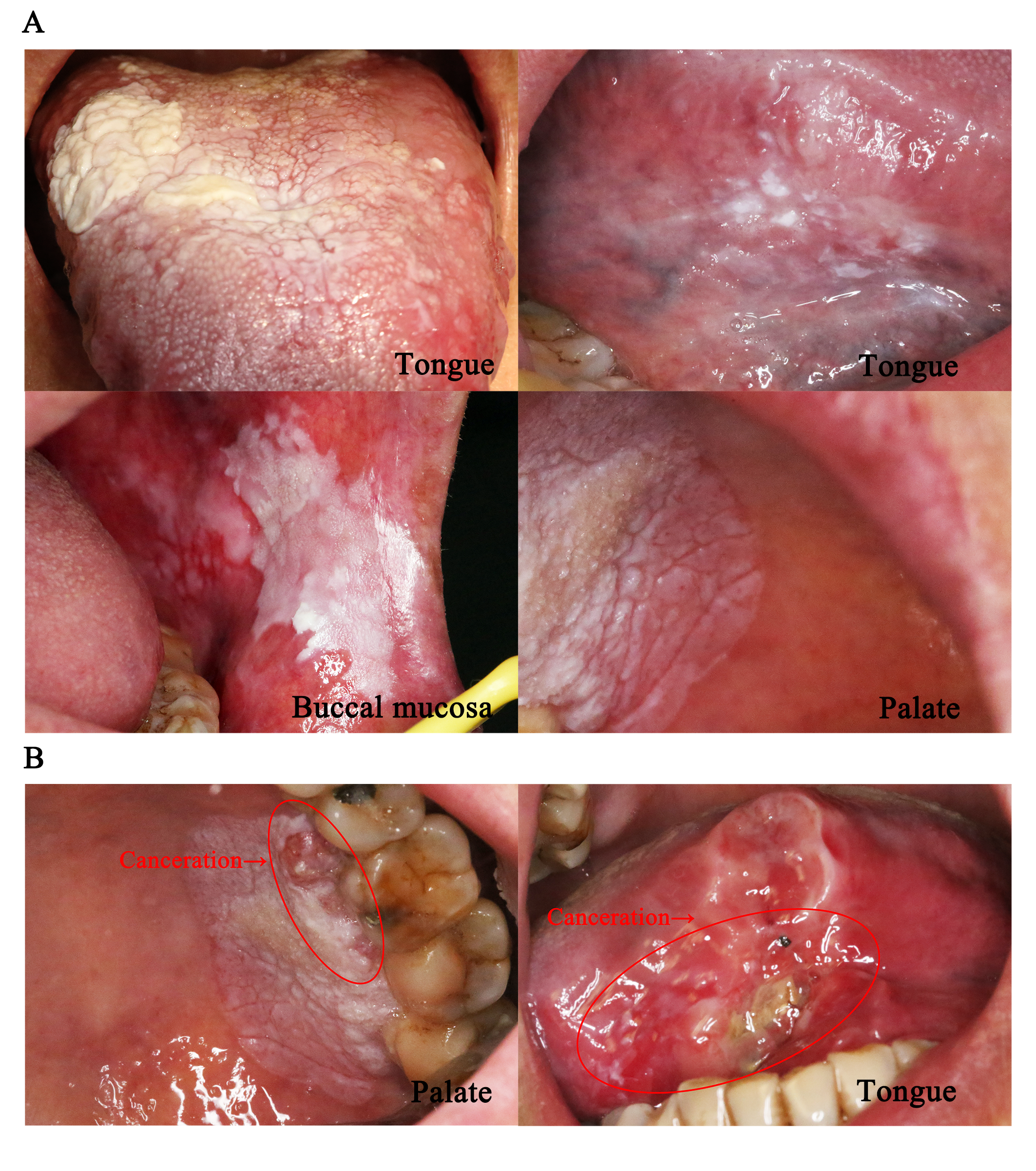

Supplement: Supplementary Figure S1 — The clinical lesion manifestation of OLK and malignant transformation of OLK. (A) The clinical feature of OLK, OLK can occurs in any part of the oral mucosa, including tongue, buccal, palate and gingiva etc. (B) The clinical manifestation of malignant transformation of OLK. [file Image_1.tif]

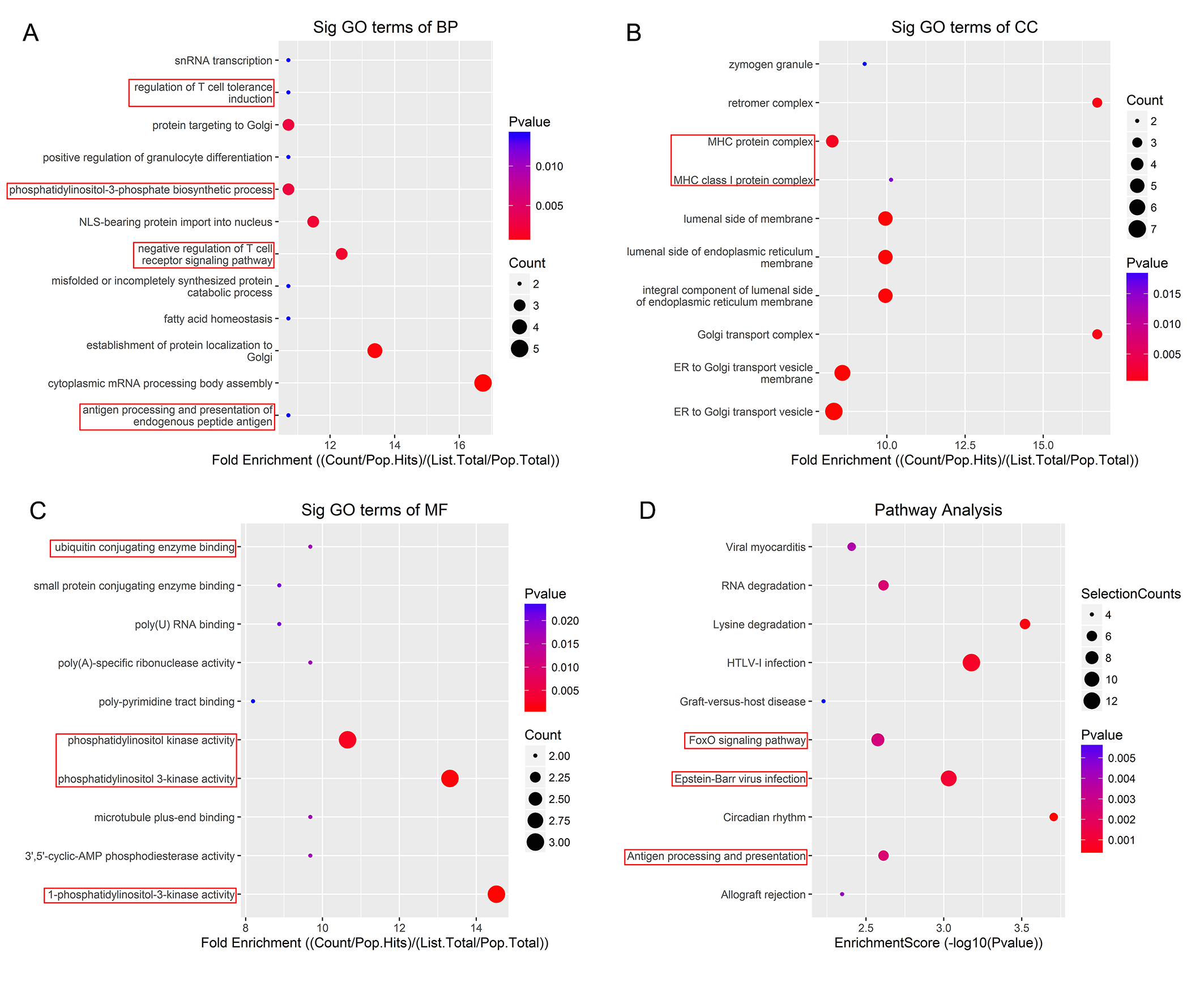

Supplement: Supplementary Figure S2 — GO enrichment and KEGG pathway analysis. (A–C) Top 10 GO terms were exhibited in BP, CC and MF bubble diagram respectively. (D) Top ten KEGG pathway involved in differentially expressed circRNAs. [file Image_2.tif]
