# Supplementary material for: Hsa_circ_0060927 Is a Novel Tumor Biomarker by Sponging miR-195-5p in the Malignant Transformation of OLK to OSCC
Source: Front Oncol. 2022 Jan 11;11:747086. doi: 10.3389/fonc.2021.747086 (PMC8786726; doi:10.3389/fonc.2021.747086)
Supplement: Supplementary file 3 [file Table_1.docx]

Table S1: Selected top 10 altered expression of circRNAs in OLK tissues by fold change(FC)

| CircRNA ID | FC | P value | | Chromosome | | | Strand | | | Gene  name | | | Catolog | sequence  length | | |
| --- | --- | --- | --- | --- | --- | --- | --- | --- | --- | --- | --- | --- | --- | --- | --- | --- |
| Top 8 upregulated circRNAs | | | | | | | | | | | | | | | | |
| hsa_circ_0060927 （chr20:52773708-52788209-） | 7.246566 | 6.08E-06 | | | Chr20 | | | - | | CYP24A1 | | exonic | | | 1106 | |
| hsa_circ_0005325 （chr19:34921481-34925873+) | 6.891147 | 3.94E-05 | | | Chr19 | | | + | | UBA2 | | exonic | | | 321 | |
| hsa_circ_0001897 （chr9:134381501-134381840+) | 6.047909 | 0.000665 | | | Chr9 | | | + | | POMT1 | | exonic | | | 158 | |
| hsa_circ_0136839 （chr8:618598-624047-) | 5.926708 | 0.00026 | | | Chr8 | | | - | | ERICH1 | | exonic | | | 954 | |
| hsa_circ_0032822 (chr14:81209419-81244390-) | 5.841145 | 0.001374 | | | Chr14 | | | - | | CEP128 | | exonic | | | 595 | |
| hsa_circ_0001730 (chr7:100410369-100410830-) | 5.719311 | 0.001192 | | | Chr7 | | | - | | EPHB4 | | exonic | | | 362 | |
| hsa_circ_0000274 (chr11:3752621-3774638-) | 4.004562 | 0.022303 | | | Chr11 | | | - | | NUP98 | | exonic | | | 556 | |
| hsa_circ_0007637 (chr16:3900298-3901010-) | 3.729912 | 0.018582 | | | Chr16 | | | - | | CREBBP | | exonic | | | 713 | |
| Top 2 downregulated circRNAs | | | | | | | | | | | | | | | | |
| hsa_circ_0042881（chr17:29483001-29509683+) | -4.945424 | | 0.034374 | | | Chr17 | | | + | | NF1 | | exonic | | 828 |  |
| hsa_circ_0029961 （chr13:33306238-33320238+) | -4.530488 | | 0.037932 | | | Chr13 | | | + | | PDS5B | | exonic | | 613 |  |
